# Virtual Screening and Bioactivity Evaluation of Novel Androgen Receptor Antagonists From Anti-PCa Traditional Chinese Medicine Prescriptions

**DOI:** 10.3389/fchem.2020.582861

**Published:** 2020-09-17

**Authors:** Wenya Han, Yuqi Shi, Jie Su, Zhennan Zhao, Xin Wang, Jiazhong Li, Huanxiang Liu

**Affiliations:** School of Pharmacy, Lanzhou University, Lanzhou, China

**Keywords:** prostate cancer, androgen receptor, traditional Chinese medicine, virtual screening, activity evaluation

## Abstract

Prostate cancer (PCa), a type of malignancy that arises in the prostate gland, is the most commonly diagnosed neoplasm and the second leading cause of cancer-related deaths in men. Acquisition of resistance to conventional therapy is a major problem for PCa patient treatment. Androgen receptor (AR) signaling pathway is necessary in the pathogenesis of prostate cancer, and there is a heightened interest in finding novel AR antagonists that target AR and its regulatory pathways. In our search for novel androgen receptor antagonists, we focus on the Traditional Chinese Medicine (TCM), which has been used for thousands of years to prove effective in the treatment of cancer. In this study, we collected 653 traditional Chinese medicine prescriptions that have certain therapeutic effect to prostate cancer, including the prescriptions and even the folk prescriptions. After summarizing the frequency of herbs and gathering the natural products contained in these prescriptions, we built a natural products database to do computer-aided virtual screening and drug-like evaluation to find potential AR antagonists. Totally 25 compounds were submitted to experimental biological activity tests. Through the MTT cell proliferation experiment, 5 chemicals were found to inhibit the proliferation of LNCaP cells in a concentration-dependent manner. Especially, MoL_11 was found to have good antagonistic activity and significantly inhibit fluorescence enzyme activity by the AR reporter gene experiment. Finally, the molecular dynamics simulation method was used to study the interaction between the most active compound MoL_11 and the wild-type and F876L mutant androgen receptor (WT/F876L AR), and it was found that F876L AR could not cause resistance to MoL_11.

## Introduction

Prostate cancer (PCa), a common non-cutaneous cancer, accounts for almost one-fifth of new cancer diagnoses, and is the second leading cause of cancer-related deaths in men in the United States (Siegel et al., 2018). According to Cancer statistics, there will be an estimated 191,930 new cases, 33,330 deaths in 2020 (Siegel et al., 2020). 5% of global deaths of prostate cancer come from China (Ferlay et al., 2015). Aging and westernization of lifestyles have increased the incidence of prostate cancer (Møller et al., 2015). The people in the developed areas and the elderly people have a higher incidence, and the survival rate of prostate cancer is not optimistic, especially in rural areas.

Androgen receptor (AR) is a ligand-dependent trans-regulatory protein that belongs to the steroid receptor in the nuclear receptor superfamily (Gao et al., 2005). Androgen receptor can be activated through the binding of endogenous androgens, such as testosterone and its metabolite dihydrotestosterone (DHT), to regulate the expression of a series of key downstream genes, causing uncontrolled proliferation of prostate cells and triggering prostate cancer (Tan et al., 2015). So AR is a highly explored target in the development and progression of PCa, regarded as the crucial therapeutic target for PCa (Brinkmann et al., 1999; Augello et al., 2014; Attard et al., 2016). AR antagonists have been commonly used to treat PCa for decades, such as flutamide, bicalutamide, enzalutamide, darolutamide, and apalutamide. These drugs have excellent effects initially, but long-term use of the drugs has resulted in the emergence of point mutations in the ligand binding domain (LBD) of AR, which eventually cause the AR to be resistant to antiandrogens, such as point mutations of T877A, W741L, and F876L etc. (Taplin et al., 1999; Hara et al., 2003; Tran et al., 2009).

Natural extracts have great potential in traditional medicines for the treatment of diseases and are also an essential resource for new drug discovery with their novel, rich and diverse chemical structures. Traditional Chinese Medicine (TCM) has been extensively used as an alternate treatment for prostate cancer. Many herbal extracts have been shown to inhibit the development of prostate cancer. Generally, TCM achieves therapeutic effects by targeting multiple physiological pathways, including AR, exerting pharmacological activities by numerous active agents and components. The complex active substances in herbs are still elusive and not accurately detected solely by routine methods (Li et al., 2015; Zhang et al., 2015).

In recent years, virtual screening (VS) has widely used in lead compound identification, which exhibits undefeatable advantage and has become very popular in drug discovery and design based on its time, cost, resources, and labor saving (Hou and Xu, 2004; Kitchen et al., 2004; Xu et al., 2014; Huang and Wong, 2016).

**Figure 1:**
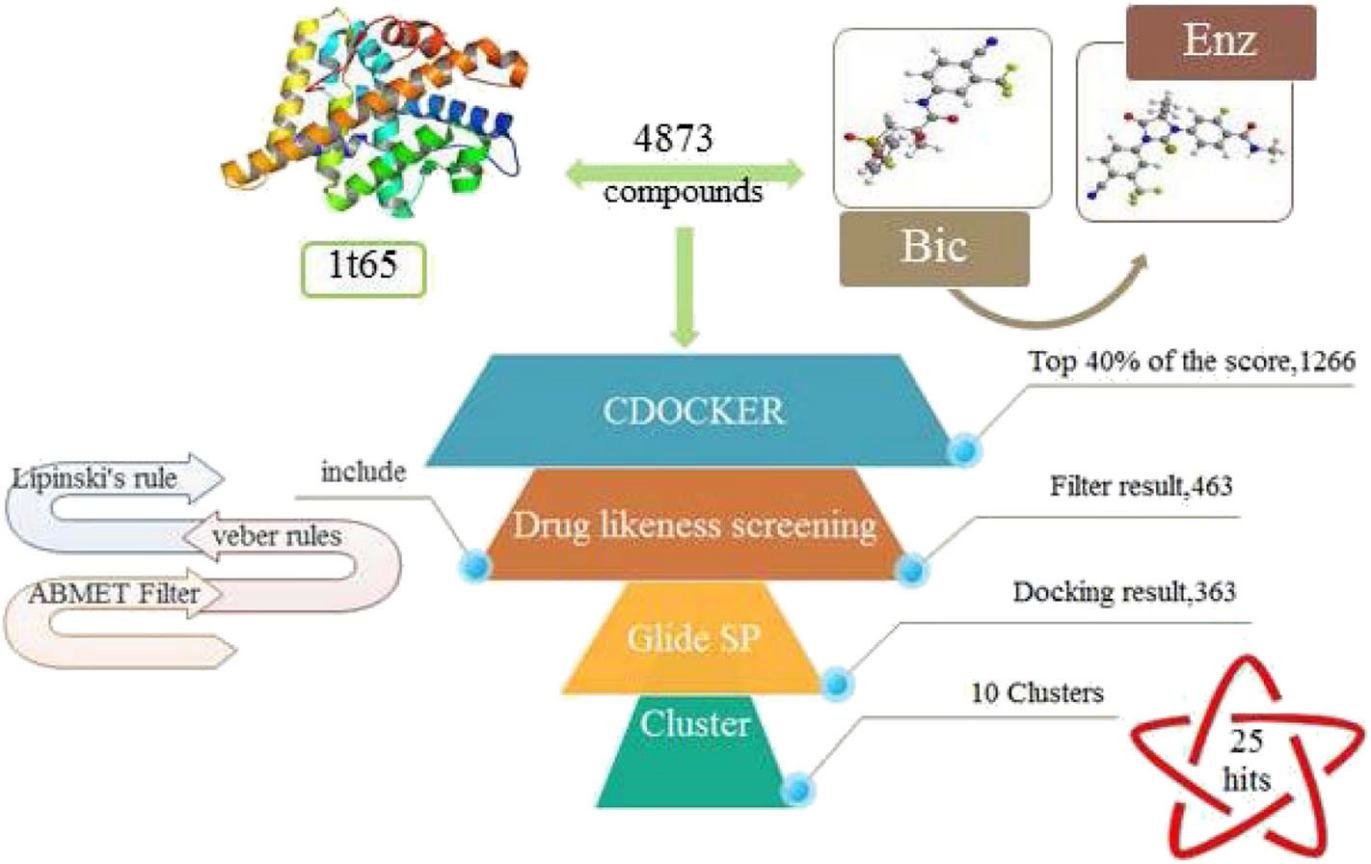
Virtual screening protocol used in this study.

In this study, we aim to screen novel AR antagonists in Chinese medicine prescriptions using virtual screening and bioassays. At the beginning, the traditional Chinese medicine prescriptions for prostate cancer were collected, and the herbs contained in them were summarized. Then, the ingredients of each herb were summarized, and the compound structure database was established. Then we conduct virtual screening protocol (Figure 1) to select potential compounds by using CDOCKER method and drug-likeness filters. The obtained 25 potential ingredients were further analyzed for bioassays, including MTT assay, AR reporter gene assay and immunofluorescence assay. The molecular simulation method was used to study the interaction mechanism between the most promising ingredient and WT 1T65/F876L AR, and the effect of F876L mutation on its AR antagonistic effect was studied.

## Materials and Methods

### Computer-Aided Virtual Screening

#### Collection of Prescriptions for PCa

The Chinese medicine prescriptions to treat prostate cancer mainly come from the books “Anti-Tumor Chinese Medicine Highlights,” “Anti-Cancer Chinese Herbal Medicine,” “Anti-Cancer Chinese Herbal Medicine and Prescription,” “Materia Medica New,” “Materia Medica,” and other books for systematic summing up prescriptions. As a result, we collected a total of 653 prescriptions.

#### Chemical Database Construction

We counted the herbs contained in the prescriptions collected above, and reserved the herbs with high frequency (≥10) for the succeeding research. Among different prescriptions, the herbs with high frequency should play important roles in anti-prostate cancer, including anti-AR effects. Then the existing chemical ingredients of each Chinese medicine were inquired by using Traditional Chinese Medicine Systems Pharmacology Database and Analysis Platform (TCMSP) (https://tcmspw.com/tcmsp.php).

#### Rough Docking Screening

The X-ray crystal structure of androgen receptor in complex with DHT (PDB entry: 1T65) was downloaded from RCSB Protein Data Bank (Estébanez-Perpi et al., 2005). The protein was prepared by using Prepare Protein tools in Accelrys Discovery Studio 2.5 software (Accelrys, 2010) and the active binding site was identified the same as endogenous androgen DHT present in the protein. Subsequently all these compounds were prepared using Prepare Ligands tools. CDOCKER is a grid-based molecular semi-flexible docking method that uses a CHARMM force field-based molecular dynamics (MD) search algorithm to dock ligands to receptor binding site (Sobhy et al., 2019). The torsions for ligands and receptors were set to be rotatable and rigid, respectively, Fast filtering was implemented using the CDOCKER protocol, the binding site radius was set to 12, the Top hits was 1, the pose was 1 and the other parameters were set as default, 3,173 small molecules docked in were obtained. The top 40% of the docked compound based on the final docking scores were retained, a total of 1,266 compounds were used for the following study.

#### Drug Likeness Screening

Initially, compounds were filtered through Lipinski's rule of five, which means that when there are more than five hydrogen-bond donors, ten hydrogen-bond acceptors, molecular weight (MWT) >500 and calculated Log P (CLogP) >5, compounds are more likely to have poor absorption or permeability (Lipinski et al., 2001). Therefore, these compounds were excluded. Subsequently, the remaining compounds were further screened by the Verb rules, which need to meet two criteria of (1) 10 or fewer rotatable bonds and (2) polar surface area equal to or <140 Å^2^ (Veber et al., 2002) to guarantee the good oral bioavailability. Ultimately, ADMET (absorption, distribution, metabolism, excretion, and toxicity) properties were used to select molecules precisely (Lagorce et al., 2008; Tian et al., 2015).

#### High Precision Screening

To further screen compounds and get more reliable docking results, Glide XP (extra Precision) docking was performed to execute the additional molecular docking. The structures of these compounds obtained above were given to the corresponding OPLS_2005 force field, and then were converted to their most probable charged states at pH 7.0 ± 2 and different tautomer were generated using the LigPrep module in Schrödinger (Schrödinger, LLC, New York, NY). The compounds with docking scores higher than enzalutamide were chosen for further analysis.

#### Novelty Analysis

The aim of this study is to obtain potential AR antagonists with rich diversity, so the hierarchical clustering method is used by the canvas module. The tanimoto coefficient based on binary fingerprints is used as a molecular similarity measure. The clustering similarity threshold was set to 0.98. Then we consider the following factors to choose suitable compounds, receptor-ligand binding mode, ligand steric hindrance, molecular skeleton, hydrocarbon group, N-heterocyclic compound, amino and amide, halogen, carboxyl, ester groups and so on.

#### MD Simulations and MM/PBSA Calculations

The wild-type androgen receptor (WT AR) 2AXA and mutant receptor F876L AR were docked with the small molecules Enz and the most promising compound, respectively, by the CDOCKER module in Discovery Studio 2.5 software. We used Pymol (http://www.pymol.org) to mutate position 876 from phenylalanine to leucine based on the WT AR to obtain mutant F876L AR. In the docking parameters, the radius of the active binding site is set to 12, and the docking pose is set to 10. Finally, the best conformation with the most optimal docking score is selected for molecular dynamics (MD) studies.

The Gaussian09 software is used to calculate the atomic charge for small molecules, and the acpype script is used to convert it into a small molecule force field file that can be recognized by the software (Sousa da Silva and Vranken, 2012), giving the protein an Amber99SB force field (Nerenberg et al., 2011). Then the coordinate files of the small molecule and protein are combined, and the topology file of the complex system is constructed. The entire system is immersed in the cube box of the spc water model (Alsharif et al., 2014). The protein is at least 1.0 nm away from the box. Na^+^ and Cl^−^ ions are added to neutralize the system charge and make the whole system neutral.

The entire molecular dynamics simulation process is completed in Gromacs 5.1.4 software (Abraham et al., 2015). After the energy of the system is minimized to ensure the stability of the protein structure, the position of the heavy atoms is restricted, the molecules around the protein are balanced, and the entire system is gradually heated from 0 to 310 K. Finally 100 ns molecular dynamics simulation analysis was conducted under 1 atm pressure and 310 K. The time step is set to 2 fs, and the track coordinates are reserved every 5,000 steps.

The combined free energy and decomposition free energy are calculated using the MM-PBSA method.

### Experimental Test and Verification

#### Cell Culture and Reagents

LNCaP (Human prostate cancer cell line) was purchased from Hangzhou Qiannuo Biotechnology Co., LTD (Hangzhou, China). Cos-7 (African green monkey kidney cell transformed with SV40) was kindly provided by Stem Cell Bank of the Chinese Academy of Sciences (Shanghai, China). LNCaP cells were propagated in RPMI 1640 medium (Hyclone) supplemented with 10% FBS and 1% penicillin—streptomycin. Cos-7 cells were grown in DMEM medium (HyClone) supplemented with 10% FBS and 1% penicillin-streptomycin. Cell cultures were maintained in a humidified atmosphere (37°C, 5% CO_2_). pcDNA3.1-AR and pMMTV-Luc were reorganized by Changsha Youbao Biological Co., Ltd. Lipofectamine3000 (Invitrogen) and Dual-Glo Luciferase Assay System (Promega) was purchased. Thiazolyl tetrazolium (MTT) was purchased from Solarbio Science& Technology. Enzalutamide (MDV-3100) was purchased from J&K. All other chemicals were obtained from Wuhan Tianzhi Biological Co., Ltd.

#### Cell Proliferation Assay

Trypsinized LNCaP cells were seeded in 96-well plates at a density of 5,000 cells per well and incubated for 24 h. When cells were attached to the bottom, the original medium was discarded, and 150 μL of serially diluted compounds solution was added which were dissolved in DMSO. After 120 h of treatment with compounds, 15 μl MTT (5 mg/ml) was supplemented to each well, and followed by another 4 h of incubation. Then 150 μl of three linked dissolved solution per well was added to dissolve the crystal of formazan for overnight (the solution was prepared from 10% SDS, 5% isobutanol and 0.01 mol/L HCl in water). Afterwards, the absorbance at 570 nm was measured by a microplate reader, and the IC_50_ values were calculated.

#### AR Reporter Gene Assay

AR-antagonist nature of test compounds was exhibited by luciferase reporter gene assay using Cos-7 cells. The Cos-7 cells were seeded in 24-well plates at a density of 40,000 cells per well and incubated for 24 h, pcDNA3.1-AR, pMMTV-Luc and pRL-SV40 vectors were co-transfected into Cos-7 cells using lipofectamine 3000 according to the manufacturer's instructions. After transfection for 24 h, cells in each well were treated with 10 nm DHT or 10 nm DHT with 10 μm compounds, and placed in the incubator for another 24 h. Then cells were harvested with 150 μL of cell passive lysis buffer, Luciferase activity was determined with luciferase assay systems (Promega) following the manufacturer's protocol to detect the AR mediated transcriptional activity.

### Statistical Analysis

The result of the experiment shows the average value of the results from three independent repeated experiments. Statistical analysis was performed using the GraphPad Prism 5.0 statistical software (GraphPad software, Inc., San Diego, CA, USA). Data are expressed as means ± standard deviation (SD). Statistical analysis was carried out using one-way ANOVA followed by Tukey's multiple comparison, with p < 0.05 considered statistically significant.

## Results and Discussion

### Statistical Analysis of TCM Prescriptions

After statistical analysis of the TCM prescriptions, we found that 82 herbs have high frequency (≥10) in the prescriptions and we summarized them in Table 1. The natural products contained in these herbs were searched and collected from TCMSP. As a result, totally 4,873 compounds were obtained to construct a 3D molecular structure database.

**Table 1:** Chinese medicine with frequency ≥10 in anti-prostate cancer prescription.

**Chinese medicine name**	
*Poria*	*Paeonia suffruticosa Andr*.
*Radix Glycyrrhizae*	*Malva verticillata* L.
*Coptis chinensis Franch*.	*Juncus effuses*
*Radix Astragali*	*Chenpi*
*Angelica sinensis*	*Eupatorium japonicum Thunb*.
*Magnolia officinalis Rehd.et Wils*	*Perotis indica (L.) Kuntze*
*Alisma orientalis (Sam.) Juzep*.	*Semen Persicae*
*Polyporus*	*Lindera aggregata (Sims) Kosterm*.
*Atractylodes macrocephala Koidz*.	*Panax ginseng C. A. Mey*.
*Cortex Phellodendri Chinensis*	*Sparganium stoloni erum, Buch. -Ham*.
*Dioscorea tokoro Makino*	*Duchesnea indica (Andr.) Focke*
*Dianthus superbus* L.	*Gardenia jasminoides Ellis*
*Solanum nigrum* L.	*Pinellia ternata (Thunb.) Breit*.
*Akebia Decne*.	*Eucommia ulmoides*
*Dioscoreae Rhizoma*	*Pyrrosia lingua (Thunb.) Farwell*
*Polygonatum sibiricum*	*Sargassum*
*Thallus laminariae*	*Carthamus tinctorius* L.
*Forsythiae Fructus*	*Ligusticum chuanxiong Hort*.
*Anemarrhena asphodeloides Bge*.	*Tetrapanax papyriferus*
*Curcuma zedoaria (Christm.) Rosc*	*Aconitum carmichaeli Debx*
*Achyranthes bidentata Blume*	*Fallopia multiflora*
*Sophora flavescens*	*Herba patriniae cum radice*
*Zingiber officinale Roscoe*	*Epimedium brevicornu Maxim*.
*Paeonia lactiflora Pall*.	*Tulipa edulis (Miq.) Baker*
*Salvia miltiorrhiza Bunge*	*Clematis chinensis*
*Polygonum aviculare* L.	*Lysimachia christinae Hance*
*Cinnamomum cassia Presl*	*Eclipta prostrate*
*Bupleurum chinense*	*Verbena officinalis* L.
*Cornus officinalis*	*Rhizoma Smilacis Glabrae*
*Radix Pseudostellariae*	*Vaccaria segetalis (Neck.) Garcke*
*Houttuynia cordata Thunb*.	*Hedyotis diffusa*
*Schisandrachinensls (Turcz.) Baill*	*Codonopsis pilosula (Franch.) Nannf*.
*Cuscuta chinensis*	*Rheum palmatum* L.
*Cistanche deserticola Ma*	*Scutellaria baicalensis Georgi*
*Morinda officinalis How*.	*Fructus Ligustri Lucidi*
*Taxillus sutchuenensis (Lecomte) Danser*	*Plantago asiatica* L.
*Scutellaria barbata D. Don*	*Lycii Fructus*
*Lobelia chinensis Lour*.	*Prunella vulgaris* L.
*Semen coicis*	*Cynanchum otophyllum*
*Kadsurae caulis*	*Lygodium japonicum (Thunb.) Sw*.
*Psoralea corylifolia* Linn.	*Rehmannia glutinosa (Gaetn.) Libosch. ex Fisch. et Mey*.

### Virtual Screening of AR Antagonists

Virtual screening techniques have become a reliable and relatively inexpensive technique for the discovery of lead compounds and have been widely used in hit identification. Docking power is a critical indicator of docking reliability for reproducing the experimental combination mode of antagonists (Tian et al., 2017). The endogenous ligand DHT was extracted from the protein 1T65 and then re-docked into the corresponding binding pocket using CDOCKER to calculate the RMSD value between the optimal pose and DHT, resulting in 0.3646 Å. below the threshold of 2.0 Å, which indicated that the docking method is reliable, and the docking protocol for virtual screening can be selected. As shown in Figure 1, the CDOCKER procedure was used at first to perform molecular docking for a rough screening of compounds. According to the -CDOCKER ENEGRY score, the top 40% chemicals with high docking scores, i.e., 1,266 compounds were retained. Subsequently, these compounds were filtered according to Lipinski's rule of five, Veber rules and ADMET Filter, and 463 molecules were chosen for subsequent analysis. Finally, the Glide XP protocol with higher docking accuracy was exploited, and 365 satisfactory compounds were harvested. Then using the Hierarchical Clustering of the Canavas module in the Schrödinger software, 10 groups of compounds were gathered. Within these clusters, 25 active ingredients were selected and purchased for subsequent bioassays (as shown in Table 2).

**Table 2:** The obtained potential ingredients by virtual screening.

**No**.	**Name**	**Structure**	**cdocker_interaction_energy**
Enz	Enzalutamide	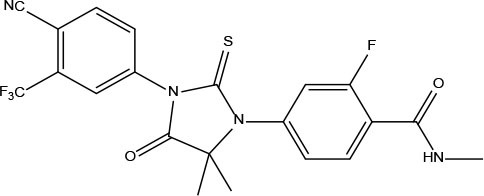	−23.6075
MOL_1	Atractylenolide III	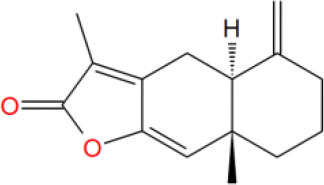	−32.3774
MOL_2	β-Caryophyllene	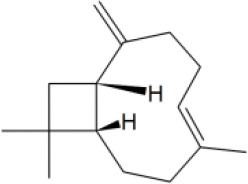	−30.8992
MOL_3	Phaseolin	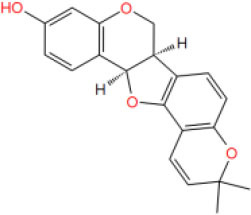	−27.8874
MOL_4	Patchouli alcohol	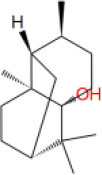	−30.2698
MOL_5	Isocurcumenol	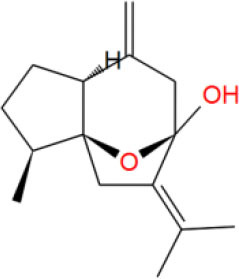	−28.2755
MOL_6	Germacrone	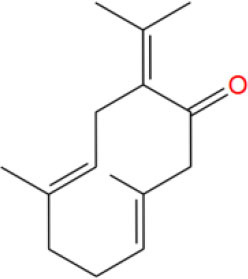	−31.3891
MOL_7	Asperuloside	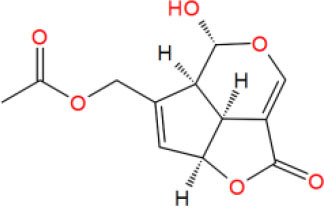	−34.1738
MOL_8	Geniposidic acid	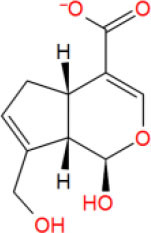	−27.5956
MOL_9	(-)-Pinoresinol	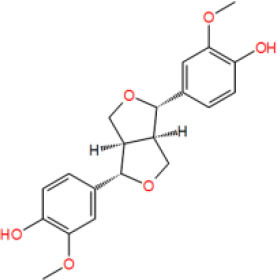	−42.336
MOL_10	Spathulenol	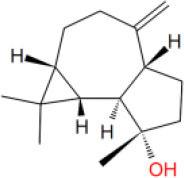	−34.1738
MOL_11	Isoimperatorin	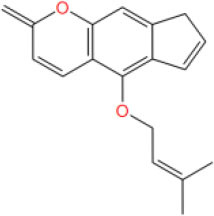	−33.9276
MOL_12	Jatrophan	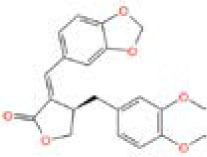	−35.1193
MOL_13	Burchellin	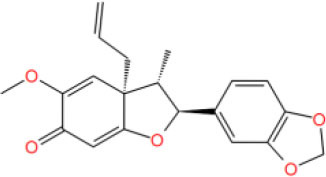	−30.0107
MOL_14	Abietic acid	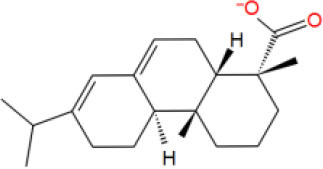	−32.697
MOL_15	Harpagoside	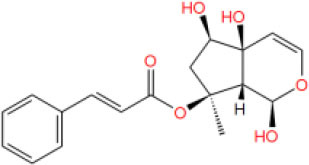	−39.4463
MOL_16	Sclareol	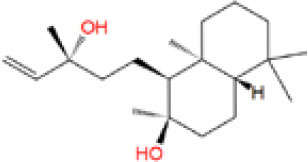	−35.1172
MOL_17	Valerenic acid	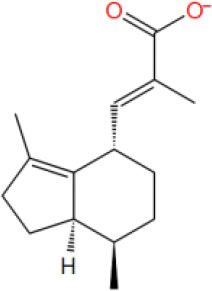	−33.652
MOL_18	Bornyl acetate	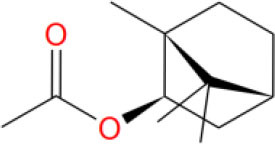	−28.9967
MOL_19	Protopine	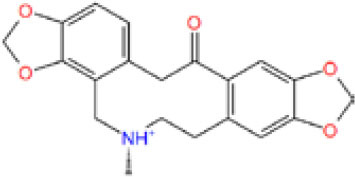	−38.5926
MOL_20	Beta-Elemene	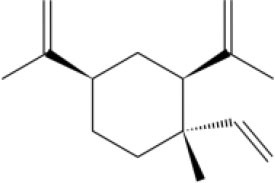	−30.1592
MOL_21	Auraptene(rg)	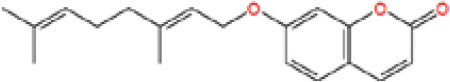	−38.5015
MOL_22	Coniferyl ferulate	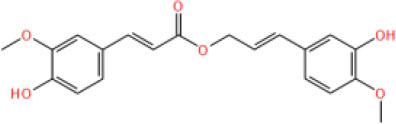	−10.0322
MOL_23	(-)-Perillyl alcohol	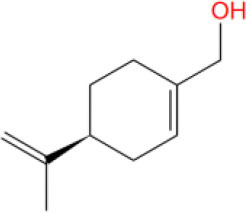	−25.1945
MOL_24	(+)-Longifolene	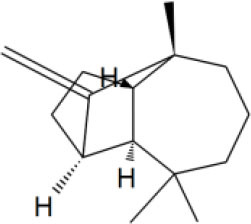	−26.5226
MOL_25	Alpha-bisabolol	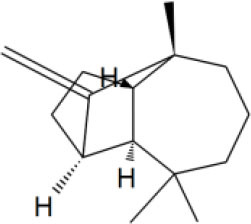	−32.6259

### Cell Proliferation Assay

The cytotoxic effect of compounds was first analyzed by MTT assay. Using enzalutamide (Enz) as the positive control, LNCaP cell was treated with different concentrations of enzalutamide and compounds for 120 h.

**Figure 2:**
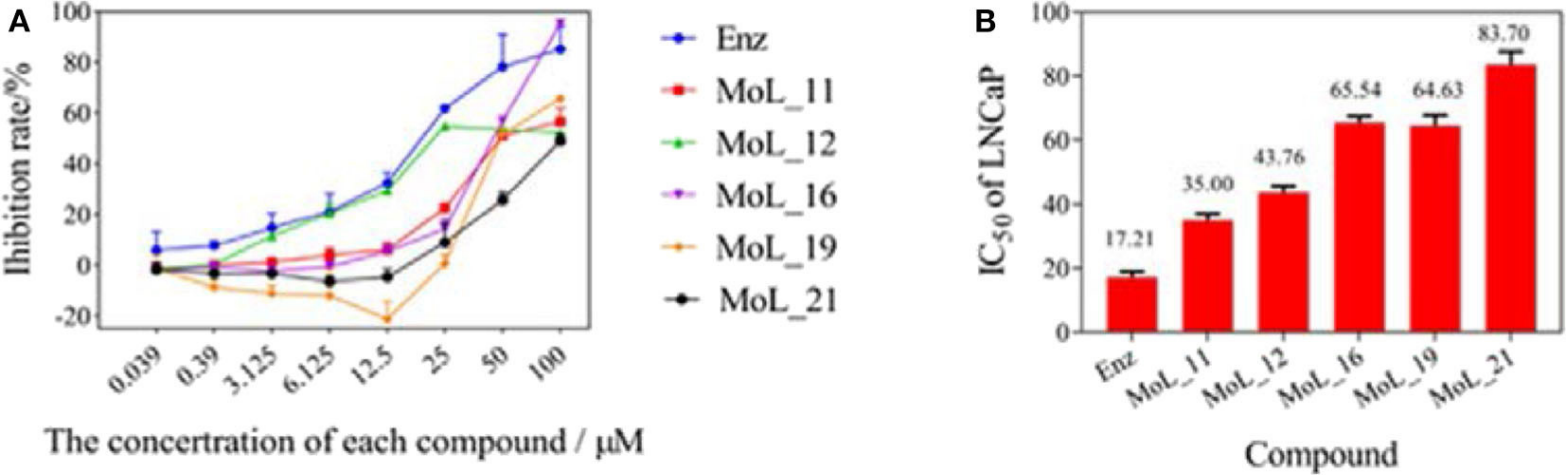
The graph of LNCaP cell inhibition activities. **(A)** is a graph showing the inhibition rate of the 6 compounds with concentration. **(B)** is the IC50 value of the 6 compounds for LNCaP.

The MTT results indicated that Enz and the five compounds can inhibit the growth of LNCaP cells in a dose-dependent manner, shown in Figure 2A. Figure 2B shows that the positive drug enzalutamide has an IC_50_ value of 17.21 μM, while the compound MoL_11 obtained by virtual screening has an IC_50_ value of 35.00 μM, which is about twice of that for Enz. Among all the tested compounds, the IC_50_ value for Mol_11 is the smallest, and the activity is relatively good. MoL_11 is derived from *Gardenia jasminoides Ellis*, also known as Isoimperatorin. The IC_50_ values of MoL_12, MoL_16, and MoL_19 increase in sequence. MoL_12 is suzilactone, which is found in *Cistanche deserticola Ma* and *Panax ginseng C. A. Mey*. MoL_16 is perillyl alcohol, present in *Salvia miltiorrhiza Bunge*. MoL_19 is the original opioid, and *Panax ginseng C. A. Mey., Cortex Phellodendri Chinensis* all have this ingredient. The IC_50_ value of MoL_21 is close to 5 times that of enzalutamide, which is orange peel olein and is present in the *Cortex Phellodendri Chinensis*.

From the cdocker interaction energy in Table 2, we can see that among the above 6 compounds with significant IC_50_ values, the most active enzalutamide has the largest cdocker interaction energy, followed by the second best activity MoL_11, then MoL_16 with the third docking value, but its activity value is in the fifth place, At the same time, MoL_21 with the worst activity value, ranked second to last in cdocker interaction energy. The best cdocker interaction energy of MoL_10 has no activity value in the MTT experiment among all the compounds in Table 2. Therefore, the docking value is not directly proportional to the final activity value, it can only be used as a preliminary judgment whether the compound has an effect on the receptor, but their effects may not be effective in biological experiments.

### AR Antagonistic Effect

The dual luciferase reporter gene can detect the expression and regulation of AR protein. PMMTV-Luc was constructed as a reporter gene plasmid, which contains 5,005 bp base pairs and 216 bp MMTV-LTR promoter sequences. MMTV-LTR is a long terminal repeat sequence of mouse breast tumors. It has four ARE sequences and can be activated as a steroid response element. pcDNA3.1-AR, pMMTV-Luc and pRL-SV40 vectors were co-transfected into Cos-7 cells, and then the dual luciferase reporter gene kit was used to detect whether the compound acted on AR.

**Figure 3:**
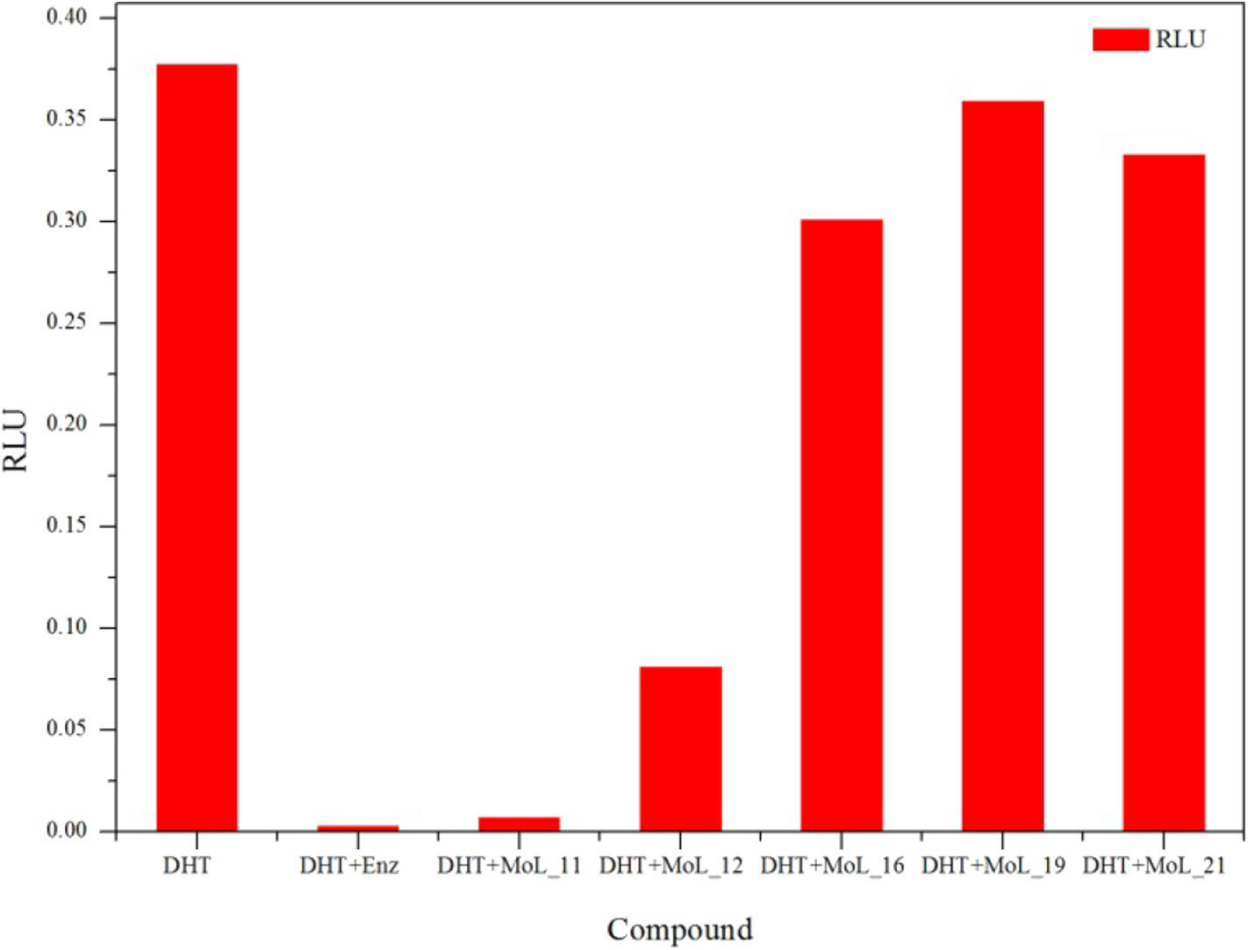
Dual luciferase gene report results.

Through the MTT cell proliferation experiment, five small molecules with significant inhibitory effects on cells were selected to study AR reporter gene experiments. DHT was used as the model group, and enzalutamide was used as the positive control group. With reference to the model group, the positive control group had a significant inhibition of AR expression, as shown in Figure 3. Among all groups, enzalutamide has the strongest antagonistic activity, and the luciferase activity decreased by 93%. The second is MoL_11, which also has obvious antagonistic activity, with an inhibition rate of 82%, indicating that MoL_11 exerts antagonistic activity through interaction with AR, rather than other toxic effects. The inhibition rate of MoL_12 is 20%, and MoL_16, MoL_19, and MoL_21 have almost no antagonistic activity, indicating that they may have an effect on cell proliferation through cytotoxicity or non-AR pathway.

### Interaction Mechanism

From the above experimental results, we know that compound Mol_11 can be a promising lead compound deserved further study. Then we conducted MD simulations to investigate the interaction of Mol_11 with AR, taking Enz as a compare. We did four different MD analyses as stated in section 2.1.7, i.e., Enz/WT AR, Enz/F876L AR, Mol_11/WT AR, and Mol_11/F876L AR.

**Figure 4:**
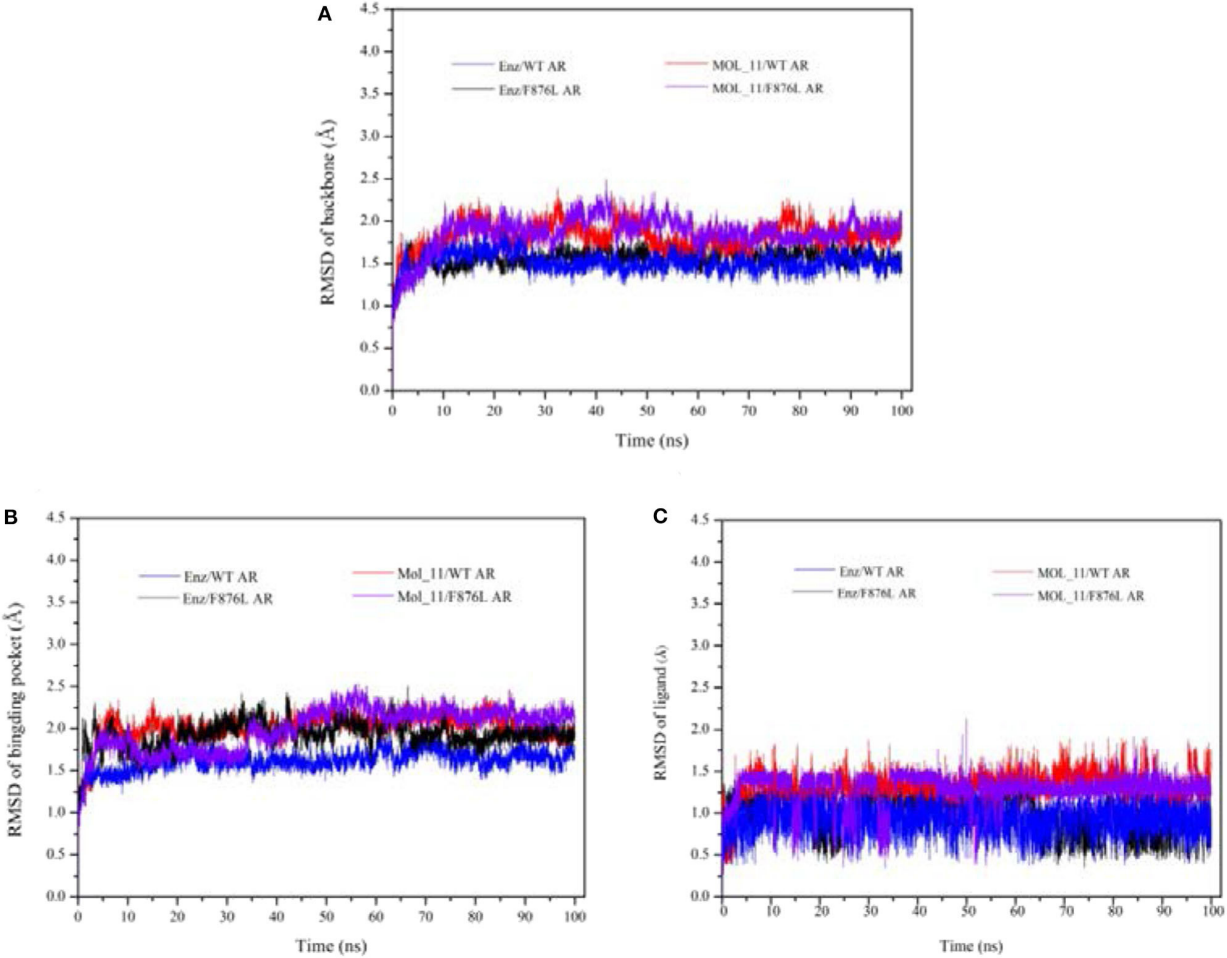
RMSD value analysis chart. **(A)** is the RMSD of the acceptor skeleton atom; **(B)** is the RMSD of the amino acid residues within 5 Å of the active pocket; **(C)** is the RMSD of the ligand. Red indicates the MOL_11/WT AR system; purple indicates the MOL_11/F876L AR system; blue indicates the Enz/WT AR system; black indicates the Enz/F876L AR system.

After 100 ns molecular dynamics simulation of the systems, the root mean square deviation (RMSD) was used to analyze and evaluate the stability of the entire simulation system. The RMSD values of the protein skeleton structure, amino acid residues within 5 Å of the active pocket and ligand atoms were calculated, as shown in Figure 4. From Figure 4A, it can be seen that the RMSD of the protein backbone of all systems became stable after 80 ns. The RMSD of the amino acid residues within 5 Å of the active pocket tends to balance after 60 ns. The RMSD of the ligand atom is also very stable and the fluctuation is small.

**Figure 5:**
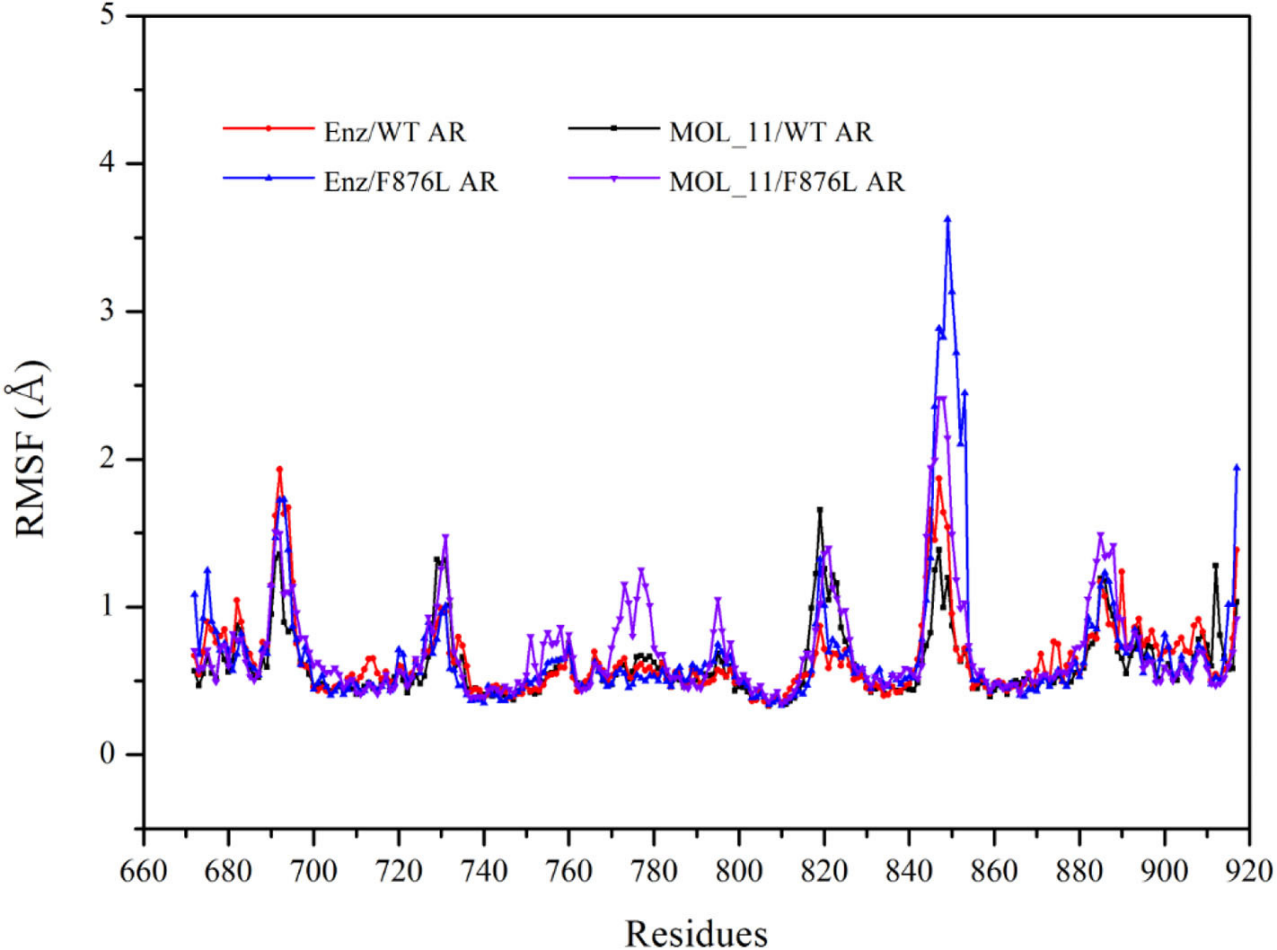
RMSF value analysis chart. Red indicates the Enz/WT AR system; blue indicates the Enz/F876L AR system; black indicates MOL_11/WT AR system; purple indicates the MOL_11/F876L AR system.

From the RMSF value analysis chart of the last 10 ns MD trajectory in Figure 5, it can be seen that RMSF value of the four systems fluctuate similarly. The MOL_11/F876L AR system fluctuates greatly in Helix-6 (H6, residues 771–778) region that obviously different from other systems. Perhaps this is the reason why MOL_11 is not resistant to F876L. In the Helix-3 (H3, residues 700–720) and Helix-5 (H5, residues 740–750) regions, the RMSF value fluctuates relatively smoothly, indicating that the amino acids in the active site of the androgen receptor are more stable than the loop region (690–695, 844–854).

Taking the RMSD of the ligand atom as the abscissa and the RMSD of the amino acid residues within 5 Å of the active pocket as the ordinate, the protein conformation was extracted through the lowest energy point to find the dominant conformation.

The α Helix-12 (H12) is a stretch of folding protein (residue 892–908 region) on the ligand binding domain of androgen receptor, and is a molecular switch for AR activation and inactivation. When AR is combined with androgen, the conformation of the H12 position changes, thereby covering the hormone-binding pocket and forming the activation functional area 2 (AF-2). This surface can recruit co-activators to promote AR transcription. If the small molecule destroys H12 and keeps it away from the binding pocket area, it is difficult to form an activation functional area, and then it will inhibit the transcription of AR and achieve the purpose of treating disease. This process is difficult to find directly in biological experiments, so it is further explained with the help of molecular dynamics simulations. Next, the action mode of the small molecule was observed through the H12 conformational change.

**Figure 6:**
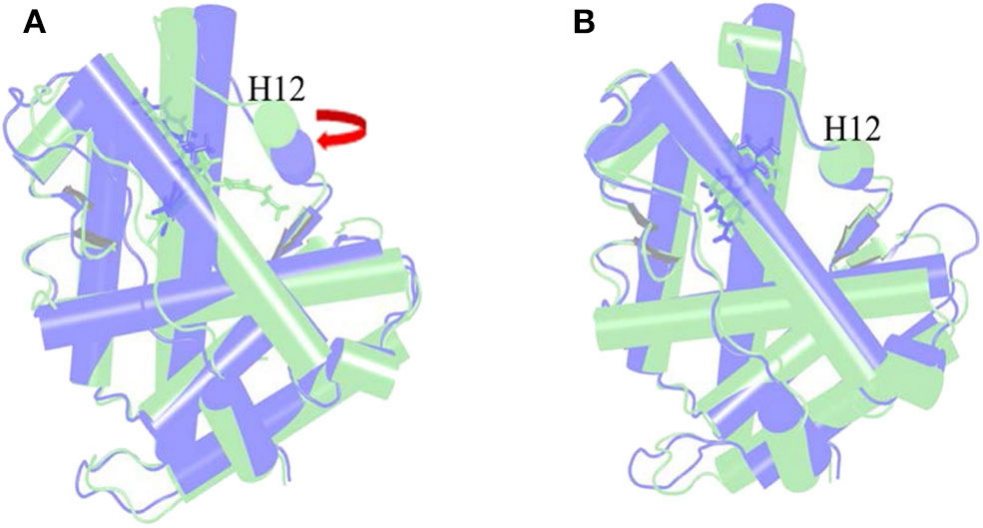
Overlay of wild type androgen receptor and mutant androgen receptor. **(A)** is the superposition of Enz wild-type system and mutant system. Green indicates the wild-type Enz/WT AR system, and blue indicates the mutant Enz/F876L AR system. **(B)** is the superposition diagram of the wild-type system and the mutant system of Mol_11. green indicates the wild-type Mol_11/WT AR system, and blue indicates the mutant Mol_11/F876L AR system.

Figure 6A is the overlay of Enz wild-type system and mutant system, green indicates the wild-type Enz/WT AR system, and blue indicates the mutant Enz/F876L AR system. We can see that the conformation of H12 has changed significantly. In the Enz/WT AR system, the Enz structure is close to H12, making it difficult for H12 to cover the binding site, resulting the disorder of AF2. In the Enz/F876L AR system, Enz is farther away from H12, which causes H12 to cover the binding site, which leads to the formation of AF2 and the drug resistance problem. It also makes Enz change from an antagonist in the original wild-type system to an agonist in the mutant system.

Figure 6B is a superposition diagram of the wild-type system and the mutant system of Mol_11. Green indicates the Mol_11/WT AR system, and blue indicates the Mol_11/F876L AR system. It can be seen from the figure that the conformation of H12 does not change, and also the conformation of Mol_11 does not change. So we can deduce that Mol_11 may not be resistant to the mutant F876L androgen receptor.

The combined free energy of each system is listed in Table 3. From the table we know that van der Waals force interaction and non-polar solvation free energy are favorable for the binding of protein and small molecules, and van der Waals force contributes most to its binding. The polar solvation free energy is not conducive to the binding. In each system, when the protein is changed from the wild type to the mutant type, the binding free energy will increase accordingly, indicating that the binding capacity of small molecules and proteins in the mutant system is stronger.

**Table 3:** Enz/F876L AR, MOL_11/WT AR, MOL_11/F876L AR, Enz/WT AR system combined with free energy analysis (unit: kcal • mol^−1^).

**Complex**	**Contribution**				
	**ΔE_**vdw**_**	**ΔE_**ele**_**	**ΔG_**p**_**	**ΔG_**np**_**	**ΔG_**bind**_**
MOL_11/WT AR	−49.197	0.734	23.850	−4.835	−29.226
MOL_11/F876L AR	−155.862	−9.438	61.042	−15.114	−119.661
Enz/WT AR	−246.292	−39.348	162.254	−22.601	−146.003
Enz/F876L AR	−245.466	−31.071	145.818	−22.619	−153.308

**Figure 7:**
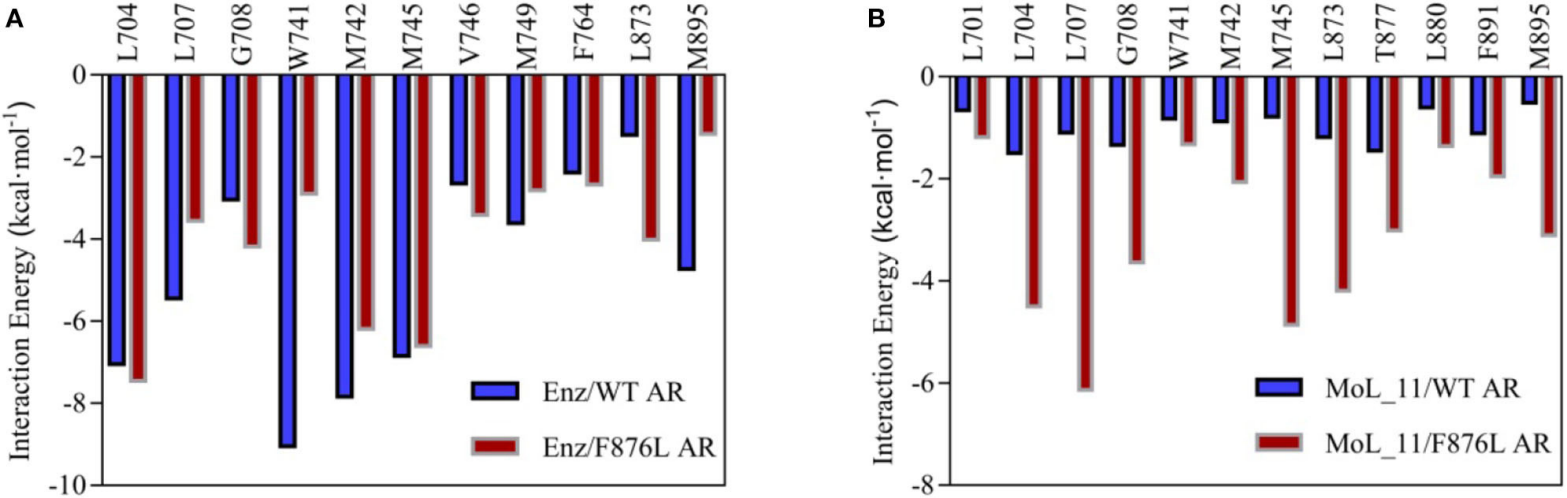
Residue energy decomposition diagram. **(A)** is the energy decomposition diagram of enzalutamide and wild-type and mutant AR residues, and **(B)** is the energy decomposition diagram of MoL_11 and wild-type and mutant AR residues (unit: kcal • mol^−1^).

The total binding free energy was decomposed to each amino acid, looking for key amino acid in the interaction between small molecules and proteins. From Figure 7A, it can be found that whether enzalutamide binds to wild-type AR or to mutant AR, the six amino acid residues L704, L707, W741, M742, M745, and M895 contribute greatly to their mutual binding. Compared with WT AR, the energy of M895 residues in F876LAR is significantly reduced (from −4.7256 kcal • mol-1 to −1.4351 kcal • mol-1), and M895 is an amino acid located on H12, which further illustrates that in the F876L AR system, the interaction between enzalutamide and H12 is weak, causing H12 to close and promote transcription, thereby promoting the conversion of enzalutamide from antagonist to agonist.

In Figure 7B, when MoL_11 is combined with F876L AR, the binding energy of all key amino acid residues increases. Among them, the binding energy of M895 amino acid residue located on H12 increases from −0.5125 kcal • mol^−1^ to −3.1034 kcal • mol^−1^, which proves that the interaction between MoL_11 and H12 is enhanced, which makes H12 deviate from the binding pocket, expose the binding site, and destroy the formation of the co-activation site, which is consistent with the analysis of the binding mode diagram of Figure 5. Therefore, MoL_11 may not cause resistance to F876L mutation.

## Conclusion

Due to the complex composition of traditional Chinese medicine, the mechanism of action is not clear. In this study, virtual screening of anti-prostate cancer traditional Chinese medicine ingredients is performed to obtain potential antiandrogens. The biological activity of the selected compounds was verified by biological experiments, and the small molecule MoL_11 was found to own the best activity. The molecular dynamics simulation method was used to study the mechanism of action between MoL_11 and wild-type AR/ F876L AR. It was found that MoL_11 will not cause resistance to F876L theoretically. The newly discovered compound, Isoimperatorin, can be a lead compound used for potential anti-PCa drug discovery.

## Data Availability Statement

The original contributions presented in the study are included in the article/supplementary material, further inquiries can be directed to the corresponding author/s.

## Author Contributions

WH undertook structure-based virtual screening and *in vitro* evaluation of hit compounds. YS and JS helped to do the *in vitro* evaluation of hit compounds. ZZ carried out molecular-dynamics simulations. XW guided the *in vitro* experiments. JL conceived and coordinated the study. HL helped to discuss the results. All authors analyzed the results and approved the final version of the manuscript.

## Conflict of Interest

The authors declare that the research was conducted in the absence of any commercial or financial relationships that could be construed as a potential conflict of interest.
